# The CILLO-E Hypothesis: Erythrocyte-Driven Acidosis and Early Eryptosis as Drivers of Cancer-Associated Anemia

**DOI:** 10.3389/fonc.2025.1697588

**Published:** 2025-10-22

**Authors:** Hüseyin Aydın

**Affiliations:** Department of Medical Biochemistry, Faculty of Medicine, Sivas Cumhuriyet University, Sivas, Türkiye

**Keywords:** erythrocyte metabolism, lactate shuttle, tumor microenvironment, cancer-associated anemia, CILLO-E Hypothesis, redox regulation, eryptosis, metabolic adaptation

## Abstract

**Background:**

The tumor microenvironment (TME) is characterized by high lactate and proton accumulation resulting from glycolytic metabolism. While acidosis is known to influence immune and stromal cells, its direct effects on erythrocytes—the most abundant circulating cells—remain underexplored.

**Methods:**

An integrative review of cancer metabolism, erythrocyte physiology, and lactate transport systems was conducted using PubMed and Web of Science. From this synthesis, the CILLO-E hypothesis (Cancer-Induced Lactate Load on Erythrocytes) was formulated.

**Results:**

The hypothesis proposes that lactate and protons enter erythrocytes via MCT1, leading to intracellular acidification. This process disrupts glycolytic enzymes, reduces ATP production, and impairs Na^+^/K^+^-ATPase and Ca²^+^-ATPase activity. Energy depletion causes Ca²^+^ overload, which activates scramblase and inhibits flippase, resulting in PS exposure and premature eryptosis. In parallel, reduced 2,3-BPG synthesis alters hemoglobin–oxygen affinity, exacerbating hypoxia. Together, these mechanisms provide a biochemical explanation for the normocytic–normochromic anemia frequently observed in cancer. Importantly, cancer-associated anemia is multifactorial, and CILLO-E should be viewed not as a comprehensive explanation but as a complementary mechanism acting through lactate-induced erythrocyte dysfunction.

**Conclusions:**

The CILLO-E hypothesis reframes erythrocytes as active metabolic targets in the TME rather than passive oxygen carriers. By linking lactate-driven metabolic stress to erythrocyte dysfunction, anemia, and systemic hypoxia, it suggests a feedback loop that promotes tumor progression and highlights opportunities for erythrocyte-based biomarkers and therapeutic strategies.

## 1 Introduction

The tumor microenvironment (TME) is a complex and dynamic ecosystem composed not only of malignant cells but also of stromal cells, immune system components, vascular structures, extracellular matrix, and diverse metabolites (1, 2). This microenvironment is shaped by cellular interactions as well as by key metabolic parameters such as pH, oxygen tension, glucose, and lactate levels (1, 3).

In recent years, the metabolic reprogramming of cancer cells—particularly the Warburg effect, glutaminolysis, and the lactate shuttle—has been recognized as a defining process of tumor progression (4, 5). In the Warburg effect, cancer cells preferentially utilize glycolysis for energy production instead of mitochondrial oxidative phosphorylation, even in the presence of oxygen (6, 7). This shift directs pyruvate toward lactate rather than acetyl-CoA, thereby enabling rapid ATP generation while simultaneously providing intermediates for biosynthetic pathways (7, 8). However, this process also leads to the accumulation of large amounts of lactate and to pronounced acidosis within the TME (9). The acidic milieu not only supports tumor cell proliferation but also promotes immune suppression, angiogenesis, and metastatic dissemination (10, 11).

In addition to the Warburg effect, cancer cells also exploit glutamine as a carbon source beyond glucose (12, 13). In the process of glutaminolysis, glutamine is converted to glutamate and subsequently to α-ketoglutarate, thereby replenishing the TCA cycle through anaplerosis. This pathway supports ATP production while contributing to the biosynthesis of nucleotides, amino acids, and lipids (5, 14). Moreover, glutamine metabolism may indirectly contribute to the maintenance of hemoglobin oxygen-carrying capacity by supporting NADPH generation, which plays a key role in counteracting oxidative stress and in the reduction of methemoglobin.

Another crucial interaction among tumor cells is the lactate shuttle. In hypoxic regions, cancer cells undergoing intensive glycolysis export lactate into the extracellular space via MCT4. Neighboring normoxic cells, in turn, import this lactate through MCT1, convert it to pyruvate, and use it as a substrate for oxidative phosphorylation. This mechanism alleviates the metabolic burden of hypoxic cells while optimizing the energy production of normoxic cells, and it also contributes partially to the stabilization of extracellular pH (15–17).

This metabolic reorganization affects not only tumor cells but also immune cells, stromal components, endothelial cells, and circulating erythrocytes. Since erythrocytes remain in continuous contact with tumor tissues across the capillary network, they may be exposed to elevated lactate and proton loads. Such exposure has the potential to profoundly impair erythrocyte functions, including NADPH-generating capacity, ion homeostasis (Na^+^/K^+^-ATPase, Ca^2+^-ATPase), and the methemoglobin reductase system, thereby compromising their oxygen delivery function (18). Importantly, cancer-associated anemia (CAA) is multifactorial; bone marrow suppression, cytokine-mediated inhibition of erythropoiesis, hemolysis, and nutritional deficiencies are among the most common causes (19–21). Within this broader context, the CILLO-E hypothesis positions lactate-induced erythrocyte dysfunction not as a comprehensive explanation but as a complementary mechanism that may significantly contribute to anemia by targeting erythrocyte metabolism and lifespan.

## 2 Gaps in the literature and aim of this study

The metabolic shift induced by the Warburg effect not only alters energy metabolism but also establishes a new biochemical equilibrium at the microscopic level within tumor tissues. In this context, while hypoxic regions reduce their metabolic burden, normoxic cells gain rapid energy flow and biosynthetic advantages (1, 6, 18). Lactate is now well recognized not merely as a metabolic by-product but also as an energy substrate and a signaling molecule (22). However, most existing studies have primarily focused on the intrinsic metabolism of cancer cells or their interactions with stromal and immune cells, leaving erythrocytes largely outside this equation.

**Figure 1:**
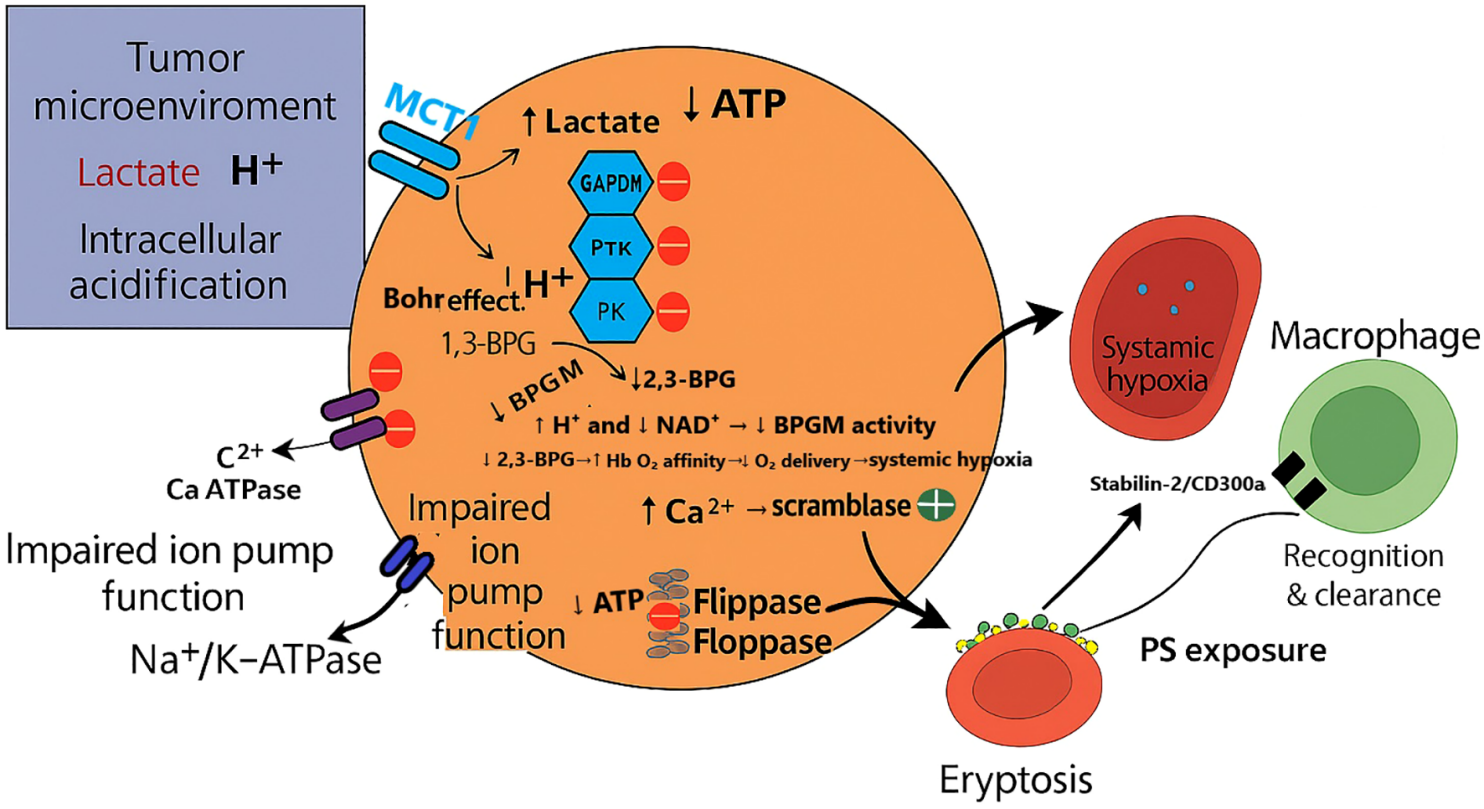
CILLO-E hypothesis: Erythrocyte dysfunction in the tumor microenvironment. The figure illustrates how lactate and protons (H^+^) generated by tumor glycolysis infiltrate erythrocytes through the MCT1 transporter, leading to intracellular acidification. This acidic stress impairs key metabolic enzymes such as GAPDH, PK, and PFK, reducing ATP production. ATP depletion, in turn, causes Na^+^/K^+^-ATPase and Ca^2+^-ATPase pump failure, resulting in ion imbalance and increased intracellular Ca^2+^ levels. Excess Ca^2+^ activates scramblase while suppressing flippase, triggering PS externalization on the erythrocyte membrane. Exposed PS acts as an “eat-me” signal for macrophages, facilitating erythrocyte clearance via eryptosis. At the same time, reduced 2,3-BPG levels and altered Hb–O_2_ affinity aggravate systemic hypoxia, which further reinforces the TME’s pathological state. This cycle highlights how erythrocyte dysfunction not only contributes to normocytic–normochromic anemia but also establishes a feedback loop that promotes hypoxia-driven tumor progression.

Yet erythrocytes, the most abundant circulating cell population, remain in constant contact with tumor tissues along the capillary network. This interaction can profoundly reshape erythrocyte metabolism, ion homeostasis, and oxygen-carrying capacity. The elevated lactate and proton load in the tumor microenvironment (TME) may not only affect immune cells but also alter erythrocyte NADPH generation through the pentose phosphate pathway, enzymatic systems responsible for reducing methemoglobin back to hemoglobin (e.g., NADH-dependent cytochrome b5 reductase), and ion pumps such as Na^+^/K^+^-ATPase and Ca²^+^-ATPase (**Figure 1**) (22, 23) Such disruptions can result in diminished oxygen delivery, enhanced oxidative stress, and shortened erythrocyte lifespan.

A rare but noteworthy clinical phenomenon, type B lactic acidosis, underscores that excessive lactate accumulation in the cancer milieu can directly influence the metabolism of circulating cells (24). Nevertheless, original studies directly investigating the effects of lactate and acidosis on erythrocyte metabolism and function remain scarce.

This study aims to address this critical gap. Our objective is to systematically elucidate how erythrocytes are affected under cancer conditions and to reposition them not merely as passive oxygen carriers but as active metabolic players in shaping the TME. By doing so, we aim to clarify how fundamental erythrocyte biochemical functions—including energy production, redox balance, oxygen release capacity, and membrane integrity—are profoundly altered by lactate accumulation and acidosis in the cancer milieu, thereby contributing both to pathophysiological understanding and to the development of potential therapeutic strategies.

## 3 Lactate metabolism and the role of MCT transporters

As highlighted in the introduction, cancer cells preferentially rely on glycolytic metabolism even in the presence of oxygen, and the large amounts of lactate produced in this process create a pronounced acidosis in the tumor microenvironment (TME). This acidic milieu imposes significant biochemical stresses not only on cancer cells but also on circulating erythrocytes.

The transcellular transport of lactate and protons is mediated by MCTs (MCTs), encoded by the SLC16 gene family (16, 25). These proton-dependent transporters function bidirectionally, facilitating both efflux and influx depending on the concentration gradient and pH differential (26). Among the isoforms, MCT1 and MCT4 are the best characterized:

MCT1 (SLC16A1): With a low Km (~3–5 mM), MCT1 remains effective even at low extracellular lactate concentrations. It is abundantly expressed in erythrocytes, endothelial cells, and muscle tissue, serving as the primary mediator of lactate/H^+^ flux in red blood cells (RBC). Under normal conditions, lactate and protons generated by glycolysis are exported from erythrocytes to plasma. However, when plasma lactate concentrations exceed the Km of MCT1, the transport direction reverses, driving lactate/H^+^ influx into erythrocytes (15, 22, 25).

MCT4 (SLC16A3): Induced by HIF-1α under hypoxic conditions, MCT4 has a high Km (~22–35 mM), enabling efficient efflux of high intracellular lactate concentrations (27).

The resulting intracellular acidification in erythrocytes leads to the inhibition of pH-sensitive enzymes, disruption of redox balance, and reduced ATP production. These consequences will be further elaborated in subsequent sections.

## 4 Red blood cell energy metabolism and cancer-associated metabolic dysregulation

### 4.1 Glycolysis and NADH functions

Since erythrocytes lack mitochondria, they rely entirely on glycolysis to meet their energy requirements. The Embden–Meyerhof pathway generates a net yield of 2 ATP and 2 NADH per glucose molecule, with lactate as the final product (28, 29).

The NADH produced serves two major functions:

- Through NADH-dependent cytochrome b5 reductase, it reduces methemoglobin back to hemoglobin, preserving oxygen-carrying capacity.
- Via lactate dehydrogenase, it reduces pyruvate to lactate, thereby regenerating NAD^+^ and ensuring the continuity of glycolysis.

These mechanisms support the production of 2,3-bisphosphoglycerate (2,3-BPG) through the Rapoport–Luebering shunt. 2,3-BPG binds hemoglobin, decreases oxygen affinity, and facilitates oxygen release to tissues (30, 31).

### 4.2 The CILLO-E hypothesis

The CILLO-E Hypothesis (Cancer-Induced Lactate Load on Erythrocytes) proposes that lactate accumulation and accompanying acidosis in the tumor microenvironment (TME) are not merely passive byproducts of metabolism. Instead, they act as active pathobiological triggers that profoundly disrupt erythrocytes at structural, metabolic, and redox levels, thereby aggravating both local and systemic hypoxia. The TME represents a stress-loaded milieu characterized by low pH, high lactate, hypoxia, an increased NADH/NAD^+^ ratio, and pro-inflammatory signals (9, 32). Erythrocytes passing through this hostile environment are impaired not only quantitatively but also in their functional, metabolic, and antioxidant capacities (17, 33).

### 4.3 Suppression of energy metabolism

Acidic pH and an elevated NADH/NAD^+^ ratio inhibit key glycolytic enzymes—phosphofructokinase (PFK), glyceraldehyde-3-phosphate dehydrogenase (GAPDH), and pyruvate kinase (PK)—thereby restricting ATP production (34). This decline in ATP availability directly compromises all energy-dependent processes previously emphasized under the section “Erythrocyte Energy Metabolism and Cancer-Associated Metabolic Dysregulation,” including ion pumping, maintenance of membrane phospholipid asymmetry, and cytoskeletal organization.

### 4.4 Ion permeability and volume regulation

Maintenance of ion homeostasis in erythrocytes is ensured by membrane pump systems, particularly Na^+^/K^+^-ATPase and Ca^2+^-ATPase. Under ATP deficiency, the efficiency of these pumps declines, leading to disruptions in ion permeability. Sodium and calcium accumulate intracellularly while potassium loss accelerates, disturbing osmotic balance and resulting in either cell swelling or, in some cases, contraction (35, 36). Moreover, elevated lactate in the acidic microenvironment increases intracellular proton load, altering the conductivity of membrane channels (37). This not only disturbs ion homeostasis but also impairs erythrocyte deformability. As ion pump activity diminishes, erythrocytes lose their flexibility, capillary transit becomes hindered, and oxygen delivery within the microcirculation is compromised (38). Consequently, energy deficiency–induced ionic imbalances emerge as critical factors that both shorten erythrocyte lifespan and contribute to hypoxia.

### 4.5 Osmotic fragility

Erythrocytes are inherently capable of adapting to diverse osmotic conditions by preserving membrane integrity. However, ATP depletion and an acidic microenvironment severely limit this adaptive capacity. Energy shortage renders ion pumps dysfunctional, resulting in sodium and water influx, potassium loss, and cell swelling (39). In swollen erythrocytes, increased membrane tension disrupts lipid asymmetry and lowers the fracture threshold of membrane skeletal proteins (e.g., spectrin, ankyrin) (40). This predisposes erythrocytes to premature hemolysis under hypotonic stress, thereby enhancing osmotic fragility. In addition, acidic conditions inhibit enzymes such as bisphosphoglycerate mutase (BPGM) and glyceraldehyde-3-phosphate dehydrogenase (GAPDH), leading to reduced 2,3-BPG levels and increased hemoglobin oxygen affinity. This paradoxically impairs oxygen delivery by reversing the Bohr effect (41). Thus, heightened osmotic fragility not only shortens erythrocyte lifespan but also worsens tissue oxygenation, further deepening the hypoxia observed in the cancer microenvironment. However, it should also be noted that in chronic hypoxia, several studies have reported an adaptive increase in 2,3-BPG that facilitates oxygen unloading (42, 43), suggesting that the net effect of lactate-induced acidosis on hemoglobin oxygen affinity is highly context-dependent.

### 4.6 Disruption of membrane phospholipid asymmetry

The erythrocyte membrane maintains its structural integrity and functional flexibility through the asymmetric distribution of phospholipids such as PS, phosphatidylethanolamine (PE), phosphatidylcholine (PC), and sphingomyelin (SM) across the bilayer. Under physiological conditions, the enzymes flippase, floppase, and scramblase act as the dynamic regulators of this asymmetry (44). However, under ATP depletion, acidic pH, and oxidative stress, flippase activity is suppressed while scramblase activity is enhanced, leading to the translocation of PS to the outer leaflet (45). The externalization of PS not only generates an “eat-me” signal for phagocytic cells but also creates a thrombogenic surface by facilitating the binding of coagulation factors (46).

Furthermore, energy deficiency limits the continuous renewal of membrane lipids, particularly reducing phosphoinositide derivatives. This disruption impairs membrane signaling and weakens interactions with cytoskeletal proteins. Consequently, erythrocytes become rigid, lose their deformability, and are more susceptible to hemolysis during passage through capillaries. In the cancer microenvironment, this process shortens erythrocyte lifespan and exacerbates functional impairments that contribute to hypoxia (47, 48).

### 4.7 Erythrocyte lifespan and hemolysis

Under normal physiological conditions, the average lifespan of erythrocytes is approximately 120 days. Despite being anucleate, this long survival is ensured by the maintenance of energy balance, preservation of membrane asymmetry, and regulation of redox homeostasis. However, in the cancer microenvironment, acidic pH, ATP depletion, NADH/NAD^+^ imbalance, and elevated oxidative stress disrupt this equilibrium, shortening erythrocyte lifespan. The externalization of PS accelerates recognition by phagocytic cells, while increased membrane rigidity and reduced deformability make erythrocytes more vulnerable to mechanical stress in the microvasculature (49). As a result, both intravascular and extravascular hemolysis are accelerated in cancer patients.

Hemolysis not only reduces erythrocyte count but also releases free hemoglobin into the plasma, which decreases nitric oxide bioavailability and thereby impairs vascular tone. Thus, the shortened erythrocyte lifespan contributes to worsening hypoxia and circulatory dysfunction (50).

### 4.8 2,3-BPG, proton accumulation, and the Bohr effect paradox

The acidic environment of the cancer microenvironment profoundly affects both glycolytic flux and erythrocyte metabolism. Acidosis inhibits the activity of BPGM, thereby reducing levels of 2,3-BPG, while the increased lactate/NADH ratio suppresses glyceraldehyde-3-phosphate dehydrogenase (GAPDH) activity, leading to reduced formation of 1,3-BPG and consequently decreased 2,3-BPG production (51). However, 2,3-BPG is one of the most important regulators facilitating oxygen release to tissues by decreasing hemoglobin’s affinity for oxygen. Therefore, a reduction in 2,3-BPG contributes to worsening hypoxia.

In parallel, increased proton concentration activates the Bohr effect, lowering hemoglobin’s oxygen affinity and enhancing oxygen release. Yet, these two processes are contradictory: while proton accumulation promotes oxygen release, the reduction in 2,3-BPG counteracts this effect by increasing hemoglobin’s oxygen affinity (41). This “Bohr effect–2,3-BPG paradox” highlights the complex regulation of oxygen metabolism in the cancer microenvironment. Consequently, erythrocytes lose functional capacity due to both energy deficiency and metabolic imbalances, failing to ensure adequate oxygen delivery to tissues.

### 4.9 Causes of anemia in cancer

The etiology of CAA is multifactorial, classically attributed to bone marrow suppression (chemotherapy-induced), nutritional deficiencies (iron, folate, or vitamin B_12_), hemolysis, decreased erythropoietin production, and the inhibitory effects of inflammatory cytokines on erythropoiesis (52). However, growing evidence suggests that erythrocyte metabolism is directly influenced by the TME. At this point, the CILLO-E hypothesis introduces a new perspective. According to this hypothesis, the acidic milieu, ATP depletion, increased NADH/NAD^+^ ratio, and impaired ion pump systems in the cancer microenvironment compromise both energy and redox homeostasis of erythrocytes. As a result, erythrocyte lifespan is shortened, membrane stability is disrupted, and oxygen transport capacity is reduced.

Therefore, one of the key contributors to cancer-related anemia may not solely originate from bone marrow suppression but also from functional impairments of erythrocytes induced by the TME. This viewpoint provides a novel biochemical framework for understanding cancer-related anemia and underscores the need for broader experimental and clinical investigations.

### 4.10 Scientific contribution and significance of the CILLO-E hypothesis

The CILLO-E hypothesis, unlike the widely recognized Warburg effect and glutaminolysis models in tumor biology, proposes that lactate is not merely an energy substrate or pH buffer but instead plays a central role in the metabolic and structural reprogramming of erythrocytes. This approach introduces three key innovations to the classical perspective on the TME.

First, the CILLO-E hypothesis shifts erythrocytes from being passive carriers to active targets in tumor biology. The reorganization of erythrocytic metabolic pathways, redox balance, and ion homeostasis in response to lactate load and acidosis emerges as a critical process that sustains a tumor-favorable microenvironment.

Second, the hypothesis emphasizes that reduced NADPH production, insufficient methemoglobin reduction, and decreased 2,3-BPG levels in erythrocytes dramatically impair oxygen transport capacity. This not only aggravates local hypoxia but also promotes systemic anemia and immune suppression, suggesting that erythrocyte dysfunction may directly contribute to immunodeficiency.

Third, the CILLO-E hypothesis reveals that erythrocyte dysfunction establishes a multilayered pathophysiological chain simultaneously affecting metabolic, hematological, and immunological processes within the TME. This chain provides wide-ranging clinical potential—from biomarker development (e.g., erythrocyte membrane PS externalization rate, NADPH/GSH levels, 2,3-BPG measurements) to erythrocyte-based targeted therapies (e.g., MCT1 inhibitors, G6PD activators, ATP stabilizers), as well as combination strategies that could be employed alongside immunotherapy or chemotherapy.

Clinical Feasibility of Proposed Biomarkers. The biomarkers proposed—PS-positive erythrocytes, 2,3-BPG, NADPH/GSH ratios, and RBC-derived microparticles—offer mechanistic insight but face translational hurdles. PS exposure and microparticles can be quantified by flow cytometry, though these assays are not yet routine in oncology (53).

2,3-BPG is measurable with enzymatic or LC-MS assays, but standardization and clinical cutoffs remain undefined (54). NADPH/GSH ratios require advanced metabolomics, currently limited to research settings (55). Importantly, these markers are not exclusive to cancer, as inflammation or hemolytic disorders may yield overlapping signatures. Hence, future work should focus on assay simplification, cost-effective platforms, and validation in tumor-specific cohorts to establish their diagnostic and prognostic value.

## 5 Materials and methods

Search Strategy and Selection Criteria: We searched PubMed and Web of Science for English-language articles published between January 2000 and August 2025 using combinations of the following terms: “TME”, “lactate”, “acidosis”, “MCT”, “MCT1”, “erythrocyte”, “RBC”, “2,3-BPG”, “eryptosis”, and “CAA”. Additional records were identified by screening reference lists. We prioritized primary research and recent reviews addressing lactate transport, erythrocyte metabolism, and anemia in cancer. Inclusion criteria were mechanistic or translational relevance to lactate/H^+^ flux and RBC function.

### 5.1 Experimental protocols required to validate the CILLO-E hypothesis

The CILLO-E (Cancer-Induced Lactate Load on Erythrocytes) hypothesis proposes that increased lactate and acidosis within TME disrupt erythrocyte metabolism, ion homeostasis, membrane asymmetry, and immune interactions, thereby contributing to anemia and hypoxia. To causally validate this mechanism, the following experimental protocols are suggested:

#### 5.1.1 Quantification of erythrocyte metabolism under acidotic–lactate loaded conditions

Under low pH and high lactate conditions, (^14^C)-lactate uptake, glycolytic enzyme activities (GAPDH, PFK, PK), ATP levels, and the NAD^+^/NADH and NADP^+^/NADPH ratios should be measured in erythrocytes. In parallel, 2,3-BPG levels and the hemoglobin–oxygen dissociation curve (p50) should be determined. This panel directly tests the core metabolic claim of the hypothesis: lactate and acidosis drive ATP/NADPH depletion, reduce 2,3-BPG levels, and shift p50 leftward, thereby producing a “paradoxical oxygenation” state that contradicts the classical Bohr effect.

#### 5.1.2 Ion pump function, volume regulation, and the Gardos axis

Under the same conditions, Na^+^/K^+^-ATPase and Ca^2+^-ATPase activities should be assessed using enzyme kinetics, while intracellular ion concentrations (Na^+^, K^+^, Ca^2+^) should be measured with ion-selective probes. Cell volume and dehydration parameters can be evaluated through flow cytometry and density fractionation. The contribution of the Gardos channel should be tested using TRAM-34, K-Cl cotransporter involvement with DIOA, and Ca^2+^ entry with BAPTA-AM, allowing causal dissection of the pathway. This approach would confirm the sequence: “ATP depletion → pump failure → Ca^2+^ increase → Gardos activation → K^+^/water loss and echinocytosis.”

#### 5.1.3 Membrane phospholipid asymmetry and eryptosis

**Figure 2:**
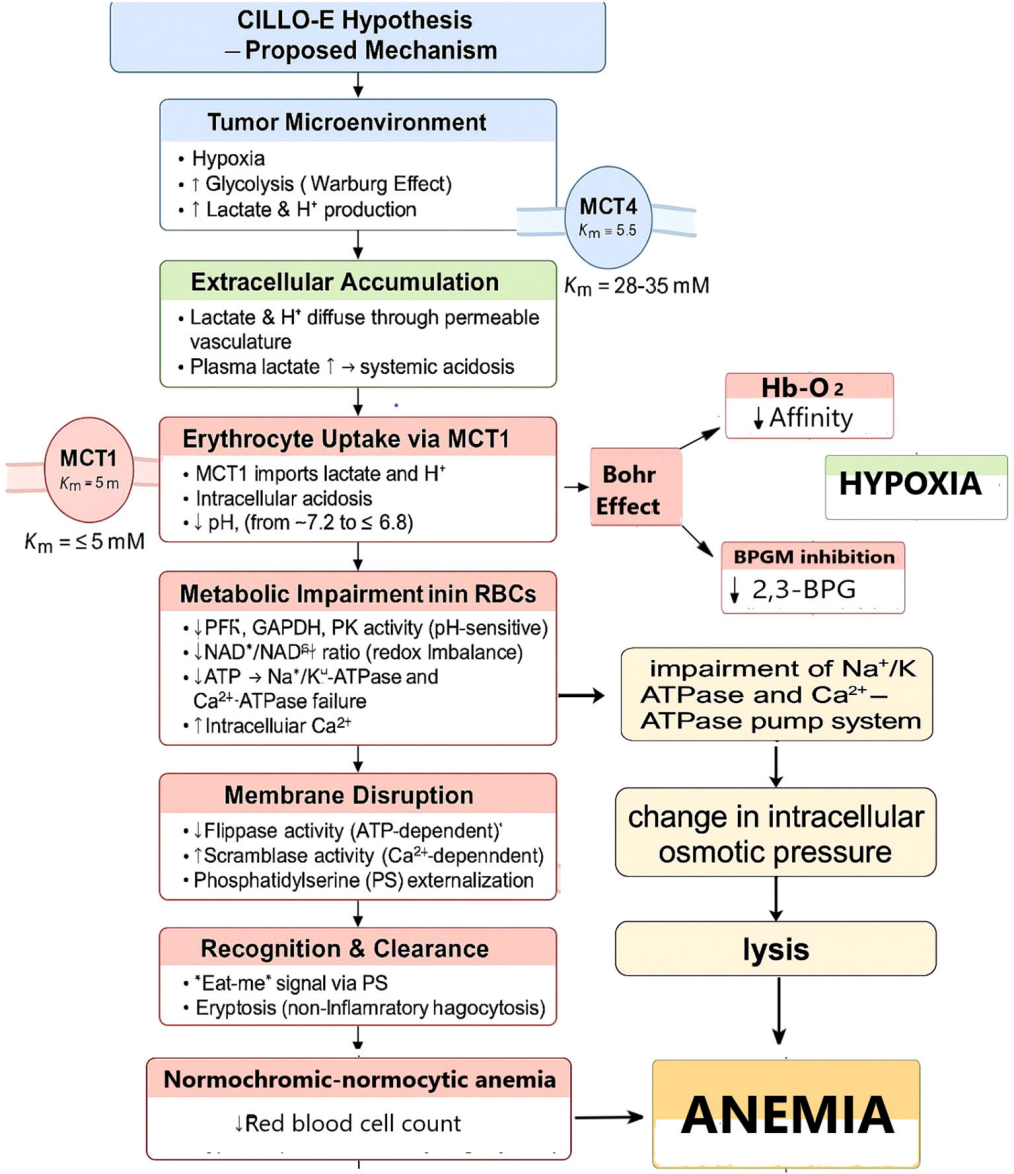
CILLO-E Hypothesis: Erythrocyte dysfunction and the mechanism of anemia in the tumor microenvironment. This proposed mechanism explains the systemic and cellular consequences of hypoxia and glycolysis-derived lactate/H^+^ production in the TME. The accumulation of lactate through vascular permeability leads to its uptake by erythrocytes via MCT1. As a result, intracellular pH decreases, metabolic enzyme activities are impaired, ATP production declines, and ion pump failure occurs. Energy depletion and elevated intracellular Ca^2+^ levels disrupt membrane integrity, causing PS externalization. This process accelerates immune recognition and clearance of erythrocytes (eryptosis). In addition, changes in Hb–O_2_ affinity and inhibition of 2,3-BPG synthesis further aggravate hypoxia. The destruction and reduction in the number of erythrocytes lead to normocytic–normochromic anemia. This model suggests that anemia in tumor biology is not only a consequence but also part of a vicious cycle that exacerbates hypoxia and fuels tumor progression.

PS externalization should be assessed by Annexin-V flow cytometry, alongside the activities of flippase and scramblase. Additionally, the number of RBC–derived microparticles (RMPs) and the percentage of PS-positive RMPs should be quantified. If PS externalization and RMP increase are demonstrated simultaneously, this would directly support the hypothesis-predicted outcomes of both procoagulant surface formation and early clearance signaling (**Figures 1**, **2**).

#### 5.1.4 Cytoskeletal integrity and deformability

Oxidative modifications and phosphorylation states of spectrin, ankyrin, and band-3 proteins should be evaluated by Western blot and mass spectrometry. Membrane surface topography can be examined using atomic force microscopy (AFM) and scanning electron microscopy (SEM). RBC deformability should be measured through laser diffraction ektacytometry and microfluidic channel assays. This would allow verification, at both structural and functional levels, of whether ATP/NADPH deficiency leads to increased cytoskeletal rigidity and loss of deformability.

#### 5.1.5 Functional analysis of erythrophagocytosis and cytokine response in macrophage co-culture

In co-cultures of PS-positive erythrocytes with primary macrophages, the rate of phagocytosis should be quantitatively assessed by confocal microscopy. Simultaneously, levels of IL-1β and TNF-α in the culture supernatant should be measured by ELISA to determine the immune response profile. To confirm experimental specificity, PS-receptor blockade assays should be performed. This methodological approach aims to test, at both structural and functional levels, the hypothesis’s dimension of “early erythrophagocytosis and immune modulation.”

#### 5.1.6 Validation with ex vivo patient samples

In erythrocytes obtained from cancer patients and healthy controls, ATP, NADPH/NADP^+^, NADH/NAD^+^, 2,3-BPG levels, metHb percentage, proportion of PS-positive cells, membrane potential (RMP), deformability, and ion contents should be measured. These parameters should be analyzed in parallel with plasma lactate concentration and pH values. This integrative assessment will establish the clinical correlation and demonstrate the hypothesis’s validity in patient biology.

#### 5.1.7 *In vivo* tumor models and intervention studies

In tumor-bearing mice, erythrocyte lifespan, 2,3-BPG levels, metHb percentage, proportion of PS-positive RBC, RMP load, and tumor oxygenation (assessed by photoacoustic imaging) should be measured. In treatment arms, MCT1 inhibition (AZD3965), pH buffering, Gardos channel blockade (TRAM-34), or pentose phosphate pathway (PPP) supporting strategies should be applied to observe whether RBC signatures are restored and whether tumor oxygenation improves.

#### 5.1.8 Multi-layered omics and network analysis

Metabolomics (glycolysis, PPP, 2,3-BPG), redox proteomics (spectrin/band-3), vesicle proteomics, and RBC-specific omics should be analyzed together to extract the multidimensional signature of erythrocyte dysfunction. This approach enables validation of the hypothesis at the systems biology scale.

#### 5.1.9 Clinical endpoints and biomarker potential

In prospective patient cohorts, plasma lactate–erythrocyte signatures–tumor oxygenation–Hb levels–and reticulocyte response should be monitored, and ROC analyses should be performed to evaluate the biomarker potential of parameters such as 2,3-BPG and PS-positive erythrocytes. This would allow testing not only the experimental but also the clinical applicability of the hypothesis.

## 6 Results

Our hypothesis suggests that tumor-derived lactate and proton accumulation profoundly alters erythrocyte metabolism, impairing enzymatic activity, ATP production, and oxygen-carrying capacity. These changes are predicted to initiate early eryptosis, reduce erythrocyte lifespan, and aggravate normocytic–normochromic anemia. Such dysfunction establishes a vicious cycle in which anemia and hypoxia reinforce tumor progression (**Figures 1**, **2**, **Table 1**).

**Table 1:** Experimental and clinical observations relevant to the CILLO-E hypothesis.

Domain	Representative finding	Context	Direction vs CILLO-E	Implication
Lactate transport	High extracellular lactate drives influx via MCT1	RBC physiology, TME gradients	Supports	Explains acidification and metabolic inhibition
pH & glycolysis	Acidosis inhibits BPGM/GAPDH → ↓2,3-BPG	*In vitro*/ex vivo	Supports	Reduced oxygen release
Hypoxia adaptation	Chronic hypoxia → ↑2,3-BPG	Exercise/tumor hypoxia	Challenges	Adaptive increase offsets acidosis effects
Ion balance	ATP depletion impairs Na^+^/K^+^-ATPase, Ca^2+^-ATPase	RBCs under metabolic stress	Supports	Leads to rigidity and hypoxia
Membrane asymmetry	PS externalization under ATP↓, Ca^2+^↑	RBC eryptosis	Supports	Early clearance, pro-coagulant activity
Clinical overlap	Type-B lactic acidosis in cancer	Case reports	Supports	Lactate burden affects circulating cells

## 7 Discussion

The CILLO-E hypothesis is not merely a theoretical model but provides a framework that can be tested through diverse experimental approaches in the laboratory. At the *in vitro* level, erythrocytes can be exposed to low pH and high lactate conditions mimicking the TME, allowing the measurement of parameters such as ATP, NADPH, 2,3-BPG, and membrane PS levels. Ex vivo approaches may include a detailed evaluation of metabolic abnormalities in erythrocytes obtained from cancer patients, with a focus on methemoglobin percentage, ion balance (Na^+^, K^+^, Ca^2+^), and redox profiles compared with healthy controls. In addition, *in vivo* animal models can be used to test lactate-reducing strategies—particularly MCT1 inhibitors (e.g., AZD3965)—for their effects on erythrocyte function and tumor oxygenation, thereby strengthening the biological validity of the hypothesis.

Several critical knowledge gaps remain in the current literature. First, the extent of decreased NADPH production in erythrocytes under TME conditions and its connection with glycolytic side branches has not been fully defined. NADPH depletion slows the reduction of methemoglobin back to hemoglobin and indirectly limits the function of energy-dependent ion pumps such as Na^+^/K^+^-ATPase and Ca^2+^-ATPase (18, 34). Therefore, under low pH–high lactate conditions, the NADPH/NADP^+^ ratio, methemoglobin percentage, and intracellular ion content should be examined using HPLC, spectrophotometry, and ion-selective electrodes, while pentose phosphate pathway flux should be confirmed by isotopic labeling techniques.

Second, changes in 2,3-BPG levels within the TME and their quantitative effects on hemoglobin oxygen affinity must be clearly established. Acidosis may decrease 2,3-BPG by altering the activities of BPG mutase or bisphosphoglycerate phosphatase, thereby reducing the p50 value and impairing oxygen release (41, 51). Thus, *in vitro* and ex vivo assays of 2,3-BPG (enzymatic assays, LC-MS), hemoglobin–oxygen dissociation curves, and simultaneous pH/lactate determinations are of critical importance.

Third, erythrocyte membrane dynamics and the mechanisms of early eryptosis remain insufficiently understood. Lactate- and acidosis-induced Ca^2+^ accumulation may trigger scramblase activation, leading to PS externalization and accelerating eryptosis (56). This process can be investigated using Annexin-V staining with flow cytometry, Western blot analysis of band 3 and spectrin degradation products, and scanning electron microscopy.

Finally, validation in *in vivo* animal models is essential for determining the clinical relevance of the hypothesis. In these models, NADPH levels, methemoglobin percentage, 2,3-BPG concentration, erythrocyte deformability, and tumor oxygen saturation should be assessed under lactate-reducing therapies. The expectation is that as lactate load decreases, erythrocyte parameters will improve and tumor growth will slow; however, compensatory mechanisms mediated by MCT4 must also be considered.

Studies supporting the hypothesis demonstrate that increased lactate in the TME disrupts erythrocyte metabolism by reducing NADPH production and limiting methemoglobin reduction (24, 41, 57). Furthermore, acidosis has been reported to decrease 2,3-BPG levels, thereby impairing oxygen release (41, 51). Similarly, Ca^2 +^ accumulation and reduced pump activity contribute to loss of deformability, while PS externalization accelerates early eryptosis and immune-mediated clearance, thereby promoting CAA (28, 56).

Nevertheless, certain findings limit the universality of the hypothesis. In particular, the MCT1-mediated lactate-buffering capacity of erythrocytes may allow pH homeostasis to be maintained even in high-lactate environments, in which case NADPH levels may not be significantly affected (25, 27). Moreover, increases in 2,3-BPG have been reported in certain hypoxic contexts, interpreted as an adaptive response that facilitates oxygen release (22). Additionally, erythrocyte responses vary depending on tumor type, stage, and host metabolic status, which further restricts the generalizability of the CILLO-E hypothesis.

Reconciling the Bohr–2,3-BPG Paradox. While acidosis reduces 2,3-BPG by inhibiting BPGM and GAPDH, studies in chronic hypoxia report an adaptive increase in 2,3-BPG that facilitates oxygen unloading (42, 43, 49). These apparently conflicting results likely reflect differences in local tumor physiology: strong acidosis favors enzymatic inhibition and 2,3-BPG decline, whereas chronic hypoxia without profound acid stress can up-regulate the Rapoport–Luebering shunt. Thus, tumor-specific context—oxygen gradients, lactate levels, and redox status—determines whether 2,3-BPG is suppressed or elevated. This variability highlights the need for standardized reporting of pH, lactate, p50, and 2,3-BPG levels in both experimental and clinical studies.

Taken together, there is strong evidence that elevated lactate and acidosis in the TME impair erythrocyte metabolism and limit oxygen-carrying capacity. However, the direction and magnitude of this effect vary according to tumor-specific factors (22, 24, 27). Therefore, rigorous validation of the hypothesis requires standardized *in vitro* models and tumor-specific *in vivo* studies.

In conclusion, the CILLO-E hypothesis proposes that lactate accumulation in the TME is not merely a metabolic byproduct but an active regulator that reprograms erythrocyte functions. This model redefines erythrocytes from passive oxygen carriers to active metabolic targets. The contribution of erythrocyte dysfunction to CAA can thus be explained not only by hematopoietic suppression but also by metabolic and structural mechanisms. In this context, MCT1 inhibitors, NADPH-enhancing or antioxidant strategies, agents supporting methemoglobin reduction, and ion homeostasis regulators represent promising therapeutic tools to preserve erythrocyte function.

## Data Availability

The original contributions presented in the study are included in the article/supplementary material. Further inquiries can be directed to the corresponding author.
